# Decreasing prevalence of no known major risk factors for cardiovascular disease among Mississippi adults, Mississippi Behavioral Risk Factor Surveillance System, 2001 and 2009

**DOI:** 10.1186/s12889-016-3860-9

**Published:** 2016-12-03

**Authors:** Vincent L. Mendy, Rodolfo Vargas, Lamees El-sadek, Vanessa L. Short

**Affiliations:** 1 Mississippi State Department of Health, Office Health Data and Research, 570 East Woodrow Wilson, Jackson, MS 39215 USA; 2 Nemours/AI duPont Hospital for Children, 833 Chestnut Street, Suite 1210, Philadelphia, PA 19107 USA

**Keywords:** BRFSS, Cardiovascular disease, Disparities, Risk factors, Mississippi

## Abstract

**Background:**

Cardiovascular disease (CVD) is the leading cause of death in Mississippi. However, the prevalence of no known CVD risk factors among Mississippi adults and the change of prevalence in the past 9 years have not been described. We assess changes in prevalence of no known CVD risk factors during 2001 and 2009.

**Methods:**

Prevalence of high blood pressure, high cholesterol, diabetes, physical inactivity, smoking, and obesity were investigated. Survey respondents who reported having none of these factors were defined as having no known CVD risk factors. Differences in prevalence and 95% confidence intervals were determined using *t*-test analysis.

**Results:**

Overall, age-standardized prevalence of having no known CVD risk factors significantly decreased from 17.3% in 2001 to 14.5% in 2009 (*p* = 0.0091). The age-standardized prevalence of no known CVD risk factors were significantly lower in 2009 than in 2001 among blacks (8.9% vs. 13.2%, *p* = 0.008); males (13.5% vs. 17.9%, *p* = 0.0073); individuals with a college degree (25.2%, vs. 30.8%, *p* = 0.0483); and those with an annual household income of $20,000–$34,999 (11.6% vs. 16.9%, *p* = 0.0147); and $35,000–$49,999 (15.2% vs. 23.3%, *p* = 0.0135).

**Conclusion:**

The prevalence of no known CVD risk factors among Mississippi adults significantly decreased from 2001 to 2009 with observed differences by race, age group, sex, and annual household income.

## Background

Cardiovascular disease (CVD) describes the major disorders of the heart and the arterial circulation supplying the heart, brain, and peripheral tissues [1]. The frequency of CVD in most populations and its association with premature death, loss of independence, impaired quality of life, social and economic costs, and health disparities make it a major public health priority [1]. Age-standardized death rates due to CVD [International Classification of Diseases (ICD), tenth revision: ICD-10, I00-I09, I11, I13, I20-I51, I60-I69, and I70] in the United States (U.S.; 312.9 deaths/100,000 population in 2001 vs 224.7 deaths/100,000 population in 2009) and Mississippi (408.7 deaths/100,000 population in 2001 vs 303.6 deaths/100,000 population in 2009) have declined over the past decade, yet CVD remains the leading cause of death both nationally and in Mississippi [2]. In 2009, Mississippi CVD death rate were higher than the U.S. rate and were disproportionately higher for blacks (354.1 deaths/100,000 population) compared to whites Mississippians (282.8 deaths/100,000 population) [2]. Considering Mississippi’s high CVD mortality rate and its disproportionate impact on the black population, preventing and reducing disparities due to CVD must remain a high priority issue for the state’s public health professionals.

One way to reduce the burden of CVD is by targeting the prevention of CVD risk factors. An increased number of Mississippi adults with no known major CVD risk factors including high blood pressure, high cholesterol, diabetes, smoking, physical inactivity, and obesity [3] can reduce associated disparities and mortality [4]. There is a critical need for primary prevention of major CVD risk factors because providing education and treating individuals with established risk factors may not effectively reduce CVD risk to a risk-level comparable to that of individuals who have never had CVD risk factors [4]. Wilkins and colleagues (2012) demonstrated that lifetime risk estimates for total CVD were high for all individuals, even those with optimal risk factors in middle age [5]. The prevalence of Mississippi adults with no known major CVD risk factors and how the prevalence of no known major CVD risk factors changed in the past 9 years have not been described.

The Centers for Disease Control and Prevention (CDC) in collaboration with the Mississippi State Department of Health, through a 5-year cooperative agreement (the Mississippi Delta Health Collaborative, MDHC), are implementing policy, environmental and systems-change interventions to target the “ABCS” (appropriate use of aspirin for those eligible, blood pressure control, cholesterol management, and smoking cessation) of heart disease and stroke prevention in the Mississippi Delta, an 18-county region of the state with high CVD burden.

To support these efforts, this study analyzes changes in prevalence of Mississippi adults with no known major CVD risk factors using 2001 and 2009 Mississippi Behavioral Risk Factor Surveillance System (BRFSS) data. In addition, prevalence changes of Mississippi adults with no known major CVD risk factors were examined by sociodemographic characteristics.

## Methods

### Data source and study population

The BRFSS is a state-based, random digit-dialed telephone survey of the U.S. non-institutionalized civilian population, aged 18 years or older. The BRFSS is conducted in all 50 states, the District of Columbia, and 3 U.S. territories (Puerto Rico, Guam, and the U.S. Virgin Islands) and has been approved by Human Research Review Boards from state departments of health. Detailed information about BRFSS is available at www.cdc.gov/brfss/.

Mississippi BRFSS data from 2001 (*n* = 3,043) and 2009 (*n* = 11,194, oversampled) were used for our analysis because all of the questions pertaining to the variables of interest were asked in these survey years. These variables include self-reported responses to questions regarding high blood pressure, high cholesterol, diabetes, current smoking, physical inactivity, and obesity. In addition, only respondents who self-identified as black or white were included in this analysis. In 2010, these racial groups accounted for 96.1% of the Mississippi population [6].

### Sociodemographic characteristics

Sociodemographic characteristics assessed included age, race, sex, annual household income, educational level, and health insurance coverage. These characteristics were categorized as: age group in years (18–34, 35–49, 50–64, ≥65); annual household income (<$20,000; $20,000–$34,999; $35,000–49,999; ≥$50,000); educational level (<high school, high school or equivalent, some college, college graduate); and health insurance coverage (yes, no), with age as a cofounding variable.

### CVD Risk factors

High blood pressure was defined as a “yes” response to the question, “Have you ever been told by a doctor, nurse or other health professional that you have high blood pressure?” High cholesterol was defined as a “yes” response to the question, “Have you ever been told by a doctor, nurse or other health professional that your blood cholesterol is high?” Diabetes was defined as a “yes” response to the question, “Have you ever been told by a doctor that you have diabetes?” Current smoking was defined as having smoked at least 100 cigarettes during the respondent’s lifetime and currently smoking at the time of the survey. Obesity was defined as having a body mass index (BMI) of 30.0 kg/m^2^ or higher (calculated from self-reported height and weight) and was categorized as obese or not obese. Physical inactivity was defined as a “no” response to the question, “During the past month, other than your regular job, did you participate in any physical activities or exercise, such as running, calisthenics, golf, gardening, or walking for exercise?” Survey respondents who reported having none of these factors were defined as having no known major CVD risk factors.

### Statistical analyses

Data were analyzed using SAS 9.3 (SAS Institute Inc.) to adjust for the disproportionate, stratified sampling design of the BRFSS and were weighted using post-stratification methods [7]. To allow for comparison between the years, the results were age-adjusted to the U.S. 2000 standard population. 95% Confidence intervals (CIs) were estimated, and differences in percentages were determined by *t*-test for the overall population and by sociodemographics characteristics using PROC DESCRIPT from SAS-callable SUDAAN 11.0.1 (RTI International). Significant differences in prevalence were determined at a *p*-value less than .05. This investigation was approved by the Mississippi State Department of Health Institutional Review Board.

## Results

Respondents’ sociodemographic characteristics were similar for survey years, 2001 and 2009. The mean age of the respondents was 44.9 and 46.3 years in 2001 and 2009 respectively. Most of the respondents were white, female, and reported having health insurance coverage. A third of the respondents reported having a high school education or equivalent and over one in five reported having some college education or are college graduates (Table 1). Overall, the age-standardized prevalence of having no known CVD risk factors significantly decreased from 17.3% in 2001 to 14.5% in 2009 (*p* = 0.0091) (Table 2). The prevalence of Mississippi adults with no known major CVD risk factors significantly decreased from 2001 to 2009 for individuals aged 45–64 years (15.0%, vs 11.3% *p* = 0.0089); blacks (13.2%, vs 8.9% *p* = 0.0089); males (17.9%, vs13.5% *p* = 0.008); persons with a college education (30.8%, vs 25.2% *p* = 0.0483); persons with an annual household income of $20,000–34,999 (16.9%, vs11.6% *p* = 0.0147) and $35,000–49,999 (23.3%, vs 15.2% *p* = 0.0135); and persons with health insurance coverage (20.0%, vs 17.1% *p* = 0.0229), respectively (Table 2).

**Table 1:** Sociodemographic characteristics of Mississippi Adults respondents, BRFSS 2001 and 2009

	2001 (*n* = 3,043)		2009 (*n* = 11,194)	
Characteristic	%^a^	95% CI	%	95% CI
Age group, years				
18–24	14.9	(13.0–16.7)	12.0	(10.6–13.4)
25–44	38.4	(36.4–40.4)	36.1	(34.6–37.6)
45–64	29.2	(27.4–31.0)	34.5	(33.3–35.8)
≥ 65	17.5	(16.1–19.0)	17.4	(16.7–18.1)
Race				
White	64.2	(62.1–66.2)	61.4	(60.0–62.9)
Black	30.2	(28.3–32.2)	34.3	(32.9–35.7)
Gender				
Female	52.9	(50.8–55.0)	52.4	(51.0–53.9)
Male	47.1	(45.0–49.2)	47.6	(46.1–49.0)
Educational level				
< High school	18.3	(16.7–19.9)	15.4	(14.4–16.5)
High school or equivalent	33.3	(31.4–35.3)	30.9	(29.6–32.2)
Some college	26.0	(24.2–27.8)	28.1	(26.7–29.4)
College graduate	22.3	(20.7–24.0)	25.6	(24.4–26.8)
Annual household income ($)				
< 20,000	23.6	(21.8–25.3)	22.5	(21.3–23.7)
20,000–34,999	24.1	(22.3–25.8)	19.6	(18.4–20.8)
35,000–49,999	14.2	(12.7–15.6)	12.2	(11.3–13.2)
≥ 50,000	19.8	(18.2–21.4)	30.8	(29.5–32.0)
No answer	18.4	(16.8–20.1)	14.9	(13.8–16.0)
Health insurance				
Yes	81.3	(79.6–83.0)	80.0	(78.7–81.4)
No	18.7	(17.0–20.4)	20.0	(18.6–21.3)

*CI* confidence interval

^a^weighted percent

**Table 2:** Percentage of Mississippi adults with no known major risk factors for cardiovascular disease by sociodemographics characteristics, Mississippi Behavioral Risk factor Surveillance System, 2001 and 2009

	2001 *N* = 459		2009 *N* = 1,187		% difference	95% CI	% relative change^b^	*p*-value^c^
	*n*	%^a^	*n*	%				
Overall	459	(17.3)	1,187	(14.5)	−2.8	(−4.82,−0.7)	16.2	0.0091
Age group (years)								
18–24	51	(24.5)	52	(23.8)	−0.7	(−10.4,8.9)	2.9	0.8786
25–44	213	(20.3)	358	(16.9)	−3.3	(−6.7,0.1)	16.3	0.535
45–64	142	(15.0)	480	(11.3)	−3.7	(−6.49,−0.9)	24.7	0.0089
≥ 65	49	(8.5)	293	(7.3)	−1.2	(−3.9,1.4)	14.1	0.3672
Race								
White	340	(18.8)	943	(17.1)	−1.6	(−4.3,1.0)	8.5	0.2275
Black	96	(13.2)	190	(8.9)	−4.3	(−7.5,−1.1)	32.6	0.008
Gender								
Female	269	(16.8)	792	(15.4)	−1.5	(−4.1,1.2)	8.9	0.2804
Male	190	(17.9)	395	(13.5)	−4.4	(−7.5,−1.2)	24.6	0.0073
Education level								
< High school	28	(6.7)	71	(4.6)	−2.2	(−5.7,1.3)	32.8	0.2185
High school or equivalent	118	(13.8)	271	(10.7)	−3.1	(−6.6,0.4)	22.5	0.0865
Some college	119	(17.5)	345	(14.2)	−3.3	(−7.1,0.5)	18.9	0.0857
College graduate	194	(30.8)	500	(25.2)	−5.6	(−11.1,0.0)	18.2	0.0483
Annual house income level ($)								
< 20,000	46	(6.4)	154	(6.9)	0.5	(−2.6,3.5)	−7.8	0.7605
20,000–34,999	112	(16.9)	195	(11.6)	−5.3	(−9.5,−1.0)	31.4	0.0147
35,000–49,999	87	(23.3)	143	(15.2)	−8.1	(−14.5,−1.7)	34.8	0.0135
≥ 50,000	153	(28.5)	543	(22.8)	−5.7	(−11.6,0.2)	20.0	0.0597
No answer	61	(13.5)	152	(10.7)	−2.8	(−7.4,1.7)	20.7	0.2239
Health insurance								
Yes	412	(20.0)	1,085	(17.1)	−2.9	(−5.5,−0.4)	14.5	0.0229
No	43	(6.8)	101	(6.7)	−0.1	(−3.1,3.0)	1.5	0.9776

*CI* confidence interval

^a^Percentages are weighted to state population estimates and age-adjusted (except those by age group) to the 2000 U.S. standard population

^b^Calculated by dividing the percentage difference between 2001 and 2009 by the percentage in 2001

^c^Determined by *t*-test

## Discussion

CVD is the leading cause of death in Mississippi and CVD rates in the state are higher than the national average [2]. The analytical results demonstrate that among adult Mississippians, the prevalence of having no known major CVD risk factors decreased significantly between 2001 and 2009. This finding is consistent with a national study that reported a similar decline in the prevalence of persons with no known risk factors for heart disease and stroke [8].

The analysis also revealed more specific patterns within this overall trend based on four sociodemographic and background variables: race, gender, socioeconomic status, and health insurance status. First, the results differed by race. Blacks, but not whites, in Mississippi experienced a significant decrease in the age-standardized prevalence of adults with no known major CVD risk factors. This finding supports a previous study that found that black adult Mississippians had a lower prevalence of ideal cardiovascular health than white adult Mississippians [9]. Further, in 2009, the age-standardized CVD death rate was 20.1% higher for black Mississippians than for white Mississippians [2]. Taken together, these findings underscore the need for culturally appropriate CVD primary prevention efforts to target black adults in Mississippi.

Second, the results of the study differed by gender. Males experienced a significant age-standardized decrease in the prevalence of Mississippi adults with no known major CVD risk factors but females did not. This result could explain previous findings that males had a higher CVD death rate than females in Mississippi [2]. Through the Barbers Reaching Out to Help Educate on Routine Screenings (B.R.O.T.H.E.R.S.) initiative, the MDHC is targeting high blood pressure and hypertension prevention among adult black males in the region [10].

Third, the results varied by socioeconomic status, which mirrors the result of previous studies [11, 12]. We found a decrease in the prevalence of having no known major CVD risk factors among participants with annual household income levels of $20,000–$34,999 and $35,000–$49,999, but not among participants with other income levels. In addition, we observed a decline in the prevalence of having no known major CVD risk factors among those with college degrees. In this analysis, higher education status was not associated with an increase in prevalence of no known major CVD risk factors. These results suggest that CVD primordial prevention programs in Mississippi, must take into account how socioeconomic characteristics shape CVD risk factor distribution in the population [13].

Finally, we found specific patterns related to health insurance status. In this analysis, we found that a majority of respondents reported having health insurance coverage, but we observed a significant decline in the prevalence of having no known major CVD among those who were insured. Previous research has shown that factors such as poor understanding of health conditions due to lack of a regular healthcare provider, cultural attitudes pertaining to healthcare systems, and lack of adherence to medical regimens influence the interaction between health insurance and CVD risk factor treatment and control [14].

The results of the current analysis shed light on previous findings and have several implications for the scholarly understanding of cardiovascular health as well as for future policy and practice in the healthcare sector. The declining prevalence of having no known major CVD risk factors may help explain the current high level of poor cardiovascular health status among Mississippi adults [9]. The results of the current study are congruent with a recent study examining CVD risk factor trends in the Mississippi Delta that found an increased prevalence of high cholesterol, obesity, and diabetes among residents [15]. In Mississippi, the prevalence of age-standardized deaths due to CVD decreased from 408.7 deaths/100,000 population in 2001 to 303.6 deaths/100,000 population in 2009 (2). However, an increase in the prevalence of CVD risk factors, coupled with a decline in the prevalence of Mississippi adults who have no known major CVD risk factors may jeopardize this decline in CVD death rates in Mississippi.

The significant decrease in the prevalence of Mississippi adults (45–64 years) with no known major CVD risk factors in the current analysis should alert Mississippi public health professionals and local healthcare policy makers of the need to direct prevention and intervention strategies toward this age group because this decrease could eventually lead to an increase in CVD mortality. In addition, the decreasing prevalence of Mississippi adults with no known major CVD risk factors calls attention to the need for primary prevention strategies to focus on preventable health risk behaviors that contribute to these CVD risk factors (high blood pressure, high cholesterol, diabetes, smoking, physical inactivity, and obesity) [4]. The 2013 American College of Cardiology/American Heart Association Guidelines on the treatment of blood cholesterol to reduce atherosclerotic cardiovascular (ASCVD) risk in adults referred to lifestyle as the “foundation” for ASCVD risk reduction [16]. These lifestyle health behaviors include adhering to a heart healthy diet, engaging in regular exercise, avoiding tobacco products, and maintaining a healthy weight [16].

Findings from this study are subject to the following limitations. First, because the BRFSS data are based on self-reports, CVD risk factor prevalence may be influenced by recall bias and the likelihood of participants offering socially desirable responses (e.g., underreporting weight, indicating that they do not currently smoke, and overstating physical activity) [17]. Second, this study did not assess preventive measures or treatment, or control for these risk factors. Third, 13.5% of participants with no known CVD risk factors did not report annual household income, and thus results based on household income may not reflect the whole population. Fourth, only 2001 and 2009 BRFSS data were assessed; we did not assess changes in risk factors between these years. Fifth, changes in the prevalence of risk factors such as diabetes and high blood pressure from 2001and 2009 may be due to changes in unawareness of these factors. Finally, we do not know whether the lower response rate in 2001 influenced the findings [8].

## Conclusion

Monitoring and evaluation of health-risk behaviors are essential to developing intervention programs, promotion strategies, and health policies that address public health at multiple levels including state, territory, metropolitan and micropolitan statistical area, and county [18]. Achieving an increase in the proportion of Mississippi adults with no known major CVD risk factors will help meet HP2020 [19] national target goals on these CVD risk factors. In addition, an increase in the proportion of Mississippi adults with no known major CVD risk factors could reduce the disproportionate high burden of CVD morbidity and mortality in the state. Community programs for CVD prevention have demonstrated favorable changes in overall CVD risk [20]. The Mississippi State Department of Health, in collaboration with CDC and other stakeholders, is currently implementing programs through the MDHC aimed at preventing and reducing heart disease, stroke, and associated risk factors in the Mississippi Delta (www.healthyms.com/MDHC). The findings of the current study can serve as a guide for local healthcare policy makers, public health professionals and stakeholders in developing intervention programs to prevent declines in cardiovascular risk and promote cardiovascular health especially among those aged 45–64 years, blacks, males, those with household incomes of $20,000–34,999 and $35,000–49,999. Future studies should examine the role of primary prevention in reducing CVD risk factors and incidence in Mississippi.

## Abbreviations

BMI: Body mass index
BRFSS: Behavioral risk factor surveillance system
CVD: Cardiovascular disease
